# Pharmacokinetic parameter driven outcomes model predicts a reduction in bleeding events associated with BAY 81–8973 versus antihemophilic factor (recombinant) plasma/albumin-free method in a Chinese healthcare setting

**DOI:** 10.1186/s12874-022-01659-w

**Published:** 2022-08-05

**Authors:** Rong Chen, Dmitry Gultyaev, Johanna Lister, Rong Han, Nan Hu, Jean Malacan, Alexander Solms, Parth Vashi, Jamie O’Hara, Shanlian Hu

**Affiliations:** 1 Hematology Department, Chengdu Third People’s Hospital, Chengdu, China; 2 Certara USA, Inc, Lorrach, Germany; 3 Formerly Certara USA, Inc, Lorrach, Germany; 4 Medical Affairs, Pharmaceuticals, Bayer Healthcare Company. Ltd, Beijing, China; 5 Global Market Access Hematology, Bayer Consumer Care AG, Peter Merian-Strasse 84, CH-4002 Basel, Switzerland; 6 Clinical Pharmacometrics, Bayer AG, Berlin, Germany; 7 Formerly at US Data Generation and Observational Studies, Bayer Corporation, Whippany, NJ USA; 8 HCD Economics, Daresbury, UK; 9 School of Public Health, Fudan University, Shanghai, China

**Keywords:** Hemophilia A, Factor VIII products, Pharmacokinetic, China, Economic model, BAY 81–8973, rAHF-PFM

## Abstract

**Background:**

Long-term prophylactic therapy is considered the standard of care for hemophilia A patients. This study models the long-term clinical and cost outcomes of two factor VIII (FVIII) products using a pharmacokinetic (PK) simulation model in a Chinese population.

**Methods:**

Head-to-head PK profile data of BAY 81–8973 (KOVALTRY®) and antihemophilic factor (recombinant) plasma/albumin-free method (rAHF-PFM, ADVATE®) were applied to a two-state (alive and dead) Markov model to simulate blood FVIII concentrations at a steady state in prophylactically-treated patients with hemophilia A. Worsening of the Pettersson score was simulated and decline was associated with the probability of having orthopaedic surgery. The only difference between the compounds was FVIII concentration at a given time; each subject was treated with 25 IU/kg every 3 days. The model used a lifetime horizon, with cycle lengths of 1 year.

**Results:**

Cumulative bleeding events, joint bleeding events, and major bleeding events were reduced by 19.3% for BAY 81–8973 compared to rAHF-PFM. Hospitalizations and hospitalization days were also reduced by 19.3% for BAY 81–8973 compared to rAHF-PFM. BAY 81–8973 resulted in both cost savings and a gain in quality adjusted life years (QALYs) compared to rAHF-PFM.

**Conclusion:**

Based on modeled head-to-head comparisons, differences in PK-properties between BAY 81–8973 and rAHF-PFM result in a reduced number of bleeding events, leading to reduced costs and increased quality of life for BAY 81–8973. These results should be used to inform clinical practice in China when caring for patients with severe hemophilia A.

**Supplementary Information:**

The online version contains supplementary material available at 10.1186/s12874-022-01659-w.

## Introduction

Hemophilia is a group of rare hemorrhagic disorders, including mutations in the factor VIII (FVIII) gene (hemophilia A). A recent systematic review and meta-analysis undertaken across China estimated that the prevalence of hemophilia A among males was 4.2 per 100,000 [1]. Patients with severe hemophilia A experience repeated spontaneous bleeding caused by the deficiency of coagulation FVIII. Frequent bleeding in the joints can lead to joint damage, deformity, disability or negatively affect patients’ quality of life [2]. Hemophilic arthropathy is the most common and severe complication observed in patients with hemophilia [3].

Prophylaxis is considered the standard of care for hemophilia patients, as it reduces the morbidity and increases the quality of life of patients [4]. Compared to on-demand treatment, prophylactic treatment can reduce joint bleeding and the development of hemophilic arthropathy [5]. Without prophylactic FVIII product replacement therapy, patients may experience repeated bleeds into the same joints, resulting in significant health-related quality of life deterioration [6].

The World Federation of Hemophilia (WFH) states that prophylaxis is always recommended over episodic therapy. While Chinese guidelines explicitly state that diagnosed patients with hemophilia A should receive treatment, with FVIII products as the recommended treatment of choice, there is currently no consensus on long-term prophylactic therapy in adult patients [7]. In countries with adequate resources, standard-dose FVIII product prophylaxis is the standard care for hemophilia patients [4]. However, in developing countries such as China, low-dose prophylactic therapy and on-demand therapy remain the primary method of treatment [3], though the mean per capita factor VIII product use in 2018 was 0.026 IU/total population, which is lower than other countries in the region with similar economic profiles [8]. While Chinese guidelines do not state a preferred prophylactic therapy, they recognize that short-term prophylaxis with high dose can reduce bleeding and improve quality of life [7].

BAY 81–8973 (KOVALTRY®) is a full-length recombinant FVIII product. A phase I, open-label, crossover study [9] compared the pharmacokinetic (PK) profile of BAY 81–8973 with antihemophilic factor (recombinant) plasma/albumin-free method (rAHF-PFM, ADVATE®) among men aged 18–65 years with severe hemophilia A and ≥ 150 exposure days to FVIII product and found that BAY 81–8973 exhibited a superior pharmacokinetic (PK) profile [9]. The PK of coagulation factors varies across populations [10]; therefore, a model integrating the long-term clinical and cost outcomes of BAY 81–8973 prophylaxis versus rAHF-PFM was developed in a Chinese population. The impacts on HRQoL, along with incremental costs, and the associated incremental cost-effectiveness ratio (ICER) are reported in order to facilitate health policy decision-making for this population.

## Materials and methods

### Model overview

**Fig. 1:**
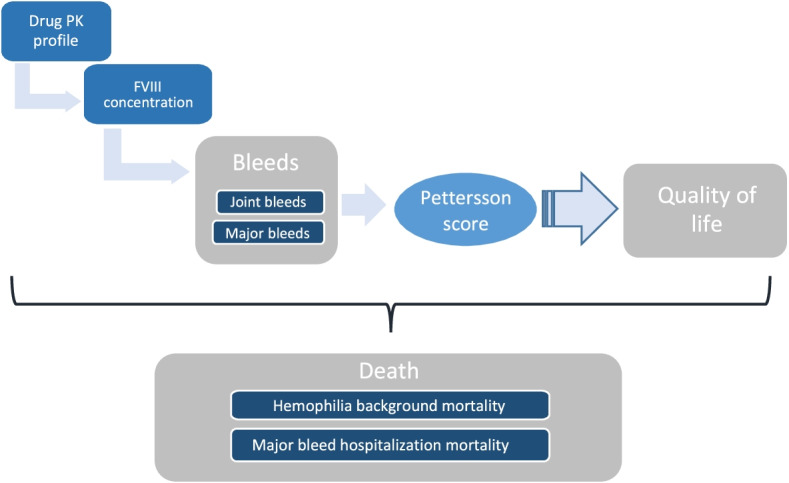
Overview of model structure

A two-state (alive and dead) Markov model was developed in Microsoft Excel to assess the cost-effectiveness of FVIII prophylaxis in adult men (≥ 18 years) with severe hemophilia A from the perspective of the Chinese health care system. The model simulates the blood FVIII concentrations at a steady state following prophylaxis treatment with either BAY 81–8973 or rAHF-PFM in a cohort of 10,000 patients. All patients entered the Markov model within the “alive” state (represented by the top half of the model structure) with the number of bleeding events determined by the model; note that within this model, bleeds are transitory and are not considered a health state (Fig. 1).

Within the alive state, patients were treated prophylactically with either BAY 81–8973 or rAHF-PFM, with the only difference between the two FVIII products being the compounds PK profile, resulting FVIII concentrations at a given time. The probability of bleeds, along with the number of bleeds, is then determined. Bleeds determine the Pettersson score, which results in the estimation of quality of life. Patients can transition to death at any time through the hemophilia background mortality. Further details on the model structure can be found in the Additional File 1. A lifetime time horizon, with cycle lengths of one year, was used. A discount rate of 5.0% was applied to costs and effects in the base case [11]. Patients were followed until death.

### Model inputs

Arthropathy development and joint replacement surgery in the model were driven by increases in the Pettersson Score, a validated radiological scoring system which ranges from 0 for joints without signs of arthropathy to a high of 78 points [12]. Patients in each bleeding-per-year category accumulated joint bleeds over time, and based on the cumulative value, the Pettersson score was determined, with an estimation of the associated joint deterioration. Patients at baseline were assumed to have had 85 joint bleeds [13] and an increase of 12.6 joint bleeds was assumed to correlate to a one point increase in the Pettersson score (95% CI: 11.1 – 14.7) [14]. A Pettersson score of 28 was assumed to represent clinically-relevant damage that required orthopaedic (joint replacement) surgery [13]. Worsening of the Pettersson score was simulated and clinically relevant decline was associated with the probability of having orthopedic surgery. Change in Pettersson score is a valid approach to projecting long-term outcomes [15].

The population-specific data on mortality in patients with severe hemophilia A is not available in China. In the absence of this information, a standardized mortality ratio was applied to the general age-adjusted mortality of men in China, based on global data of hemophilia A patients having a life expectancy of about 10 years less than the general population [16]. A sensitivity analysis assuming equivalent life expectancy between treated hemophilia A patients and that of the general population was also explored [17]. To account for the increased risk in death following major surgery or bleed, the probability of death in the year following major orthopaedic surgery or major bleed was increased by an additional 1% [18].

Utility values were health-state specific and are presented in Table 1.

**Table 1:** Utility and cost values, base case analysis

**Utilities**		
State	Mean (SD)	Source
Pettersson score 0–4	0.82 (± 0.13)	Fischer, K. et al.[6]
Pettersson score 5–12	0.81 (± 0.12)	
Pettersson score 13–21	0.77 (± 0.13)	
Pettersson score 22–39	0.74 (± 0.12)	
Pettersson score 40–78	0.72 (± 0.11)	
Orthopaedic surgery	-0.39 (± 0.04)	Ballal, R. D. et al.[19] Utility decrement is assumed to last for 30.4 days. Standard deviation not reported, 10% assumed
Hospitalisation due to major bleed	-0.39 (± 0.04)	Assumption. Utility decrement is assumed to be the same as for orthopaedic surgery but for a shorter time period (3.6 days)
**Costs**		
Input	Mean (CNY)	Source
BAY 81–8973 per IU	4.488	Huo et al.[20]
rAHF-PFMper IU	4.488	Huo et al.[20]
Hospitalization for major bleed	74,122	Expert opinion, based on survey
Physician visit	500	Expert opinion, based on survey
Cost of orthopaedic surgery	300,465	Expert opinion, based on survey
Rehabilitation costs	937.50	Expert opinion, based on survey
Total compensation per hour	25.40	National Bureau of Statistics of China[21]
**Resource Use**		
Input	Unit/Frequency	Source
Extra dose for treatment of bleed – BAY 81–8973	24.11 IU/kg	Calculated
Extra dose for treatment of bleed – rAHF-PFM	24.55 IU/kg	Calculated
Doctor visits (with or without joint bleeds)	12 / year	Expert opinion
Retirement age	60	China labour statistics[22]
Full time employment	45.6%	Sun et al.[23]
Missing days of work, annual	3.40	Chen et al. 2017[24]

rAHF-PFM: antihemophilic factor (recombinant) plasma/albumin-free method

The utility associated with arthropathy was based on the Pettersson score [12]. Major bleeds associated with hospitalisation were assumed to have the same utility deterioration as for orthopaedic surgery. The duration of a major bleed was assumed to be 3.6 days which was validated by a Chinese clinical expert involved in the study herein.

Direct costs in the model included costs of the following: prophylaxis treatment, hospitalisation for treating major bleeds, cost of physician visit (with or without bleeds), and cost of orthopaedic surgery (Table 1). The dose assumed in the model was *25 IU/kg every 3 days*.

In absence of published data, costs for hospitalization for major bleed, physician visits, orthopaedic surgery and rehabilitation were taken from a survey conducted in 2016 among five physicians in five different hemophilia treatment centers that were selected across four major cities in China (Beijing in North China, Guangzhou in South China, Chengdu in West China and Wuhan in Central China). Lost productivity due to missing work was included in the model. This was calculated based on the estimated proportion of males who were employed, the estimated annual number of days missed of work per year, and the average total compensation per hour. Unit costs are reported in 2020 Chinese yuan (CNY).

### Outcomes

Based on differences in PK between BAY 81–8973 and rAHF-PFM, the model evaluated number of bleeding events, total costs and quality-adjusted life-years. The ICER was calculated as the total incremental costs divided by the total incremental quality-adjusted life years. The base case analysis is presented from the public payer perspective; scenario analyses from a societal perspective, accounting for indirect costs due to the productivity losses experienced by both patients and caregivers, is also presented.

Both a probabilistic and deterministic sensitivity analyses were undertaken to assess parameter uncertainty. Multiple scenario analyses were considered including varying the dosing to 20 IU/kg and 15 IU/kg, given the lower doses use observed in China [25], varying the number of joint bleeds at baseline (lower value of 42.5 and upper value of 170), as well as a separate source of utility values identified in a hemophilia A review from the Institute of Clinical and Economic Review given the sparse amount of data available in China specifically and the relevance of US frameworks in other country contexts [26].

## Results

### FVIII concentration

**Fig. 2:**
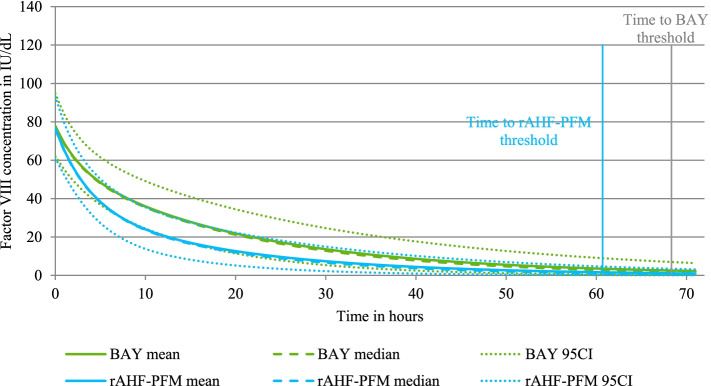
FVIII concentrations over time 95CI: 95% confidence interval; rAHF-PFM: antihemophilic factor (recombinant) plasma/albumin-free method; BAY: BAY 81–8973

The FVIII concentrations (with their 95% prediction intervals) are presented in Fig. 2.

As the elimination phase was longer for BAY 81–8973 with a longer half-life, FVIII concentrations were maintained above 1 IU/dL over a longer period of time. Time to achieve 1 IU/dL were 68.3 h (95% CI 52–72) and 60.7 (95% CI 40–72) after dosing for BAY 81–8973 and rAHF-PFM, respectively.

### Bleeding events

**Fig. 3:**
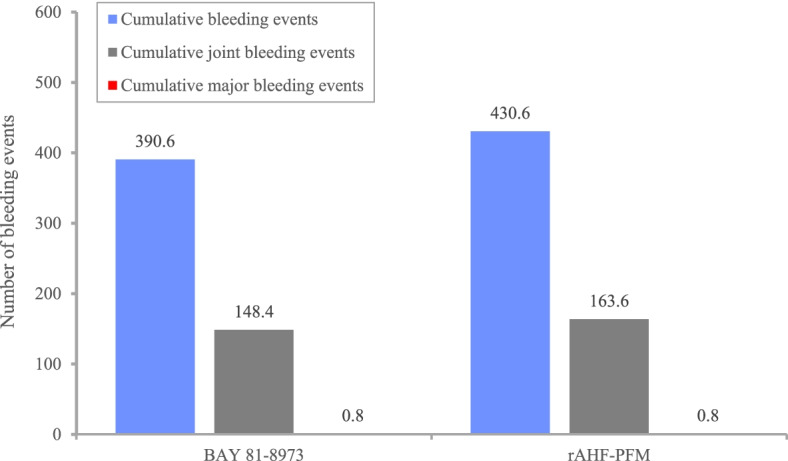
Bleeding events rAHF-PFM: antihemophilic factor (recombinant) plasma/albumin-free method; BAY: BAY 81–8973

Assuming a baseline of 85 joint bleeds, cumulative bleeding events, cumulative joint bleeding events and cumulative major bleeding events, over 12 months, are presented in Fig. 3 for BAY 81–8973 and rAHF-PFM.

Cumulative bleeding events, joint bleeding events, and major bleeding events were reduced by 19.3% for BAY 81–8973 compared to rAHF-PFM.

### Hospitalizations and orthopaedic surgeries

There were a total of 0.3 hospitalizations with a mean of 0.3 hospitalization days for BAY 81–8973 compared to 0.4 hospitalizations with a mean of 0.4 hospitalization days for rAHF-PFM over a lifetime. This represents a reduction in hospitalizations of 19.3% for treatment with BAY 81–8973 compared to rAHF-PFM. Orthopaedic surgeries were equally lower for BAY 81–8973 versus rAHF-PFM: 0.05 vs 0.07, representing a decrease of 31.6%.

### Pettersson score

Average Pettersson score increased with increasing age, over the lifetime of the cohort, but was lower for those receiving BAY 81–8973 (10.9) compared to rAHF-PFM (12.3).

### Incremental cost-effectiveness results

Costs and QALYs by health state are presented in Table 2.

**Table 2:** Cost-effectiveness results, base case analysis

	**BAY 81–8973**	**rAHF-PFM**	**Incremental**
Drug/Procedure cost	¥16,329,777	¥16,325,648	¥4,129
Bleeding related costs	¥486,877	¥612,852	-¥125,974
Physician visits	¥108,348	¥108,321	¥27
Societal cost	¥5,388	¥5,390	-¥2
**Total cost**	**¥16,930,390**	**¥17,052,210**	**-¥121,819**
QALYs	13.220	13.167	0.053
Life years	18.058	18.053	0.005
**ICER**	**BAY 81–8973 is dominant (lower costs and higher QALY gain)**		

*ICER* Incremental cost-effectiveness ratio, rAHF-PFM: antihemophilic factor (recombinant) plasma/albumin-free method; QALYs: quality-adjusted life year

Prophylactic treatment with BAY 81–8973 resulted in a cost savings of ¥121,819, mainly due to savings in bleeding related costs over the total lifetime of the patient. BAY 81–8973 compared to rAHF-PFM also resulted in 0.053 incremental QALYs. This resulted in BAY 81–8973 being a dominant strategy over rAHF-PFM (less costly and more effective).

### Sensitivity analyses

**Fig. 4:**
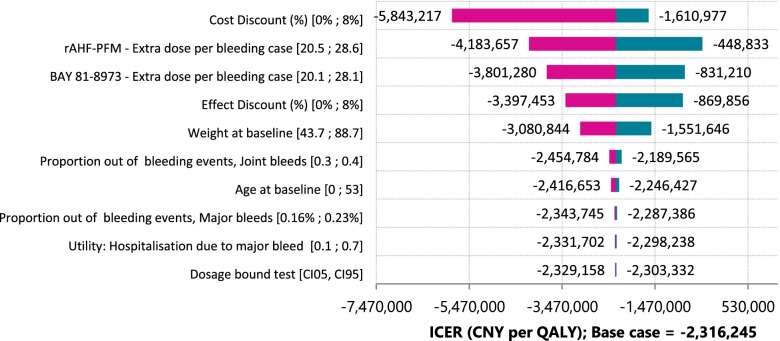
Tornado diagram indicating the effect of each independent variable on the resultant ICER while keeping all other variables constant rAHF-PFM: antihemophilic factor (recombinant) plasma/albumin-free method

Figure 4 presents the one-way deterministic sensitivity analyses. The model was most sensitive to utility values for Pettersson scores 13 – 21 and 22 – 39, followed by discounting.

When all parameters were varied simultaneously, BAY 81–8973 was less costly and cost-effective in over 95% of the scenarios compared to rAHF-PFM.

Scenario analyses of using lower prophylaxis dosing every three days were explored. As dosing decreased from the base case of 25 IU/kg to 20 IU/kg and 15 IU/kg, BAY 81–8973 remains cost-effective compared to rAHF-PFM as presented (Table 3); however, the absolute effectiveness of BAY 81–8973 is greatest with the highest dose.

**Table 3:** Scenario analyses of lower dosing

	**Dosing 25 IU/kg (base case)**	**Dosing 20 IU/kg**	**Dosing 15 IU/kg**
**Costs**			
BAY 81–8973	¥16,930,390	¥13,703,193	¥10,487,104
rAHF-PFM	¥17,052,210	¥13,821,831	¥10,599,846
**Incremental cost**	**-¥121,819**	**-¥118,638**	**-¥112,742**
**QALYs**			
BAY 81–8973	13.220	13.202^a^	13.179^a^
rAHF-PFM	13.167	13.151^a^	13.132^a^
**Incremental QALYs**	**0.053**	**0.050**	0.047
**ICER**	**BAY 81–8973 is dominant (lower costs and higher QALY gain)**		

^a^Derived using the annual bleeding rate of 25 IU/kg

*ICER* incremental cost-effectiveness ratio, rAHF-PFM: antihemophilic factor (recombinant) plasma/albumin-free method; QALYs: quality-adjusted life years

Varying the number of joint bleeds at baseline or the utilities had negligible impact on the results compared to the base case analysis.

## Discussion

Hemophilia A results in substantial morbidity and the World Federation of Hemophilia recommends management with prophylaxis as part of standard of care. Given individual variability in response to treatment of FVIII concentrates in patients with hemophilia A, PK-tailored prophylaxis is being proposed and considered as a more efficient approach for treatment with FVIII products. This study assessed the impact of prophylactic treatment with BAY 81–8973 and rAHF-PFM on health-related quality of life and costs for patients with severe hemophilia A in China, using a PK model. This model found that prophylactic treatment with BAY 81–8973 resulted in cost savings given the reduction in bleeding. Therefore BAY 81–8973 is a cost-effective option as compared to rAHF-PFM, across all doses; however, total QALY gain and incremental QALYs were higher with the highest dose of 25 IU/kg every 3 days.

These results are aligned with previous PK studies, where BAY 81–8973 was shown to have better PK characteristics, as measured by slower clearance, compared to rAHF-PFM [27]. A PK modelling study undertaken in China in pediatric patients found that annual bleeding rates and annual joint bleeding rates decreased in patients switching from rAHF-PFM to BAY 81–8973 [28]. Similar clinical outcomes were observed in adult patients, that found that the annual bleeding rate, and the annual joint bleeding rate was reduced with prophylactic treatment with BAY 81–8973 [29]. The incremental QALY gain in this study of 0.02 is also found to be greater than that identified in a cost-effectiveness models of other prophylactic treatments vs standard of care [26].

Relationship between actual FVIII concentration and bleedings were analyzed by means of a repeated time to event approach based on data obtained in the clinical development program of BAY 81–8973 (Leopold I [30]) given 20–50 IU/kg 2–3 times per week for the duration of the study. In this regard, simulations beyond the dose range investigated and the observed treatment duration have less validity. For instance, long-term treatment effects (e.g. improvement of joint status) beyond the effect of actual FVIII level on bleeding risk could not be described based on the underlying data. However, as this is assumed to equally impact treatment with BAY 81–8973 and rAHF-PFM, the overall conclusion won’t be affected by such limitations.

This study has limitations. The presented results focus on a constant treatment over the lifetime of a patient of patients receiving prophylaxis. Episodes of non-compliance or changes to a given treatment schedule over time, which is likely more reflective of a real-world patient experience, were not considered. However, as such variations in the treatment schedule would affect both treatment arms, the model outcomes would be affected equally and the omission of this does not impact the results observed. To that end, this model does not explore the impact of prophylaxis vs no prophylaxis. In the absence of data from on-demand treatment or placebo, this study is not able to estimate the connection between FVIII and bleeding corresponding to untreated patients; these results cannot be applied to treatment strategies other than prophylaxis. Further, no covariates were included in the model to adjust for differences in patient baseline characteristics. The yearly bleeding rate was also assumed to remain constant over time. In order to explore the scenarios of lower doses, the bleeding rate in the population receiving 25 IU/kg was used as a proxy for 20 IU/kg and 15 IU/kg as dose ranges were not extensively studied in the LEOPOLD trial [30]; scenario analyses of lower doses should be interpreted with caution. The analysis also assumed equivalent PK/PD relationships between BAY 81–8973 and rAHF-PFM resulting in the cost differences being driven solely by the PK differences. Given the lack of clinical head-to-head data on the products, there is limited data to confirm or deny this assumption. Our assumption was based on prior research estimating the EC50 values are identical and that the effectivity of the compounds do not change (therefore differences are PK driven) [31, 32]. This assumption is also deemed conservative since the bleeding rates for BAY 81–8973 were lower as compared to rAHF-PFM with the same dosing schedule favoring neither product.

Finally, every effort was made to source inputs in a Chinese population, however, this was not always available. Inputs that may not be generalizable to a Chinese population include the utilities used in this model, the proportion of bleeds that occurred in the joints and the proportion of bleeds that were considered major.

## Conclusion

This is the first cost-effectiveness model of adult patients with hemophilia A in China treated prophylactically with FVIII product. Given the high cost of caring for severe hemophilia A patients with a chronic, prophylaxis treatment, optimizing the costs and benefits of various treatment approaches is of paramount importance to payers in China. This PK model demonstrated that BAY 81–8973 (KOVALTRY®) is a cost saving and cost effective option as compared to rAHF-PFM (ADVATE®), due to reductions in bleeding events along with an increase quality of life, highlighting the value of BAY 81–8973 for prophylactic treatment of patients with severe hemophilia A in China.

## Supplementary Information

**Additional file 1: Table S1.** BAY 81-8973 and rAHF-PFM PK parameters(7)**. Figure S1.** Cost-effectiveness scatterplot.

## Data Availability

All data generated or analysed during this study are included in this published article and therefore, all methods were performed in accordance with the relevant guidelines and regulations. There is no additional data to be disclosed or shared.
