# Supplementary material for: Pharmacokinetic parameter driven outcomes model predicts a reduction in bleeding events associated with BAY 81–8973 versus antihemophilic factor (recombinant) plasma/albumin-free method in a Chinese healthcare setting
Source: BMC Med Res Methodol. 2022 Aug 5;22:215. doi: 10.1186/s12874-022-01659-w (PMC9356410; doi:10.1186/s12874-022-01659-w)
Supplement: Supplementary file 1 — Additional file 1: Table S1. BAY 81-8973 and rAHF-PFM PK parameters(7). Figure S1. Cost-effectiveness scatterplot. [file 12874_2022_1659_MOESM1_ESM.docx]

**Supplementary Methods**

FVIII concentration-time profiles for all patients for prophylactic treatment with BAY 81-8973 and rAHF-PFM were simulated according to Shah et al. (1), who studied PK data in an intra-individual cross-over study comparing the PK of BAY 81-8973 and rAHF-PFM in adult patients with severe hemophilia A by means of a population approach. Each subject was treated with a 25 IU/kg every 3 days, as per regimens recommended by Chinese guidelines (2). The PK profile of both compounds were described using a two-compartment model; within a patient, the only PK parameters that differed between BAY 81-8973 and rAHF-PFM were the elimination (CL) and peripheral volume of distribution (V_2_) (**Table S1**) (1). A 2x2 matrix was included in the simulation. 10,000 patients were simulated in the PK part of the model for one year and then patient results were grouped into categories by the number of bleeds: 0-2, 2-4, 4-6, 6-8, 8-10, 10-12, 12+. The average number of bleeding events per category per year was determined, with the proportion of patients in each category over time. Joint bleeds were accumulated within each bleeding category per year, separately. The bleeding rate at a given time was then calculated in the model based on FVIII concentration and cumulative risk. For each simulation subject the instantaneous bleeding risk as function of the FVIII concentration was simulated over time (3). Based on standard principles of time-to-event analysis, the bleeding risk was translated into an expected yearly bleeding rate. Abrantes et al. then studied the association between patients’ FVIII concentration and observed bleeding events based on the clinical development program of BAY 81-8973(4) by means of an repeated-time-to-event approach (3). It was found that the instantaneous bleeding risk is significantly associated with the FVIII level and the bleeding risk increases with decreasing concentration of FVIII, as described by the following formula:

$$h\left( t \right)=\lambda\cdot e^{\gamma\cdot\left( t-1 \right)}.\left( 1-\frac{{FVIII(t)F}_{VIII}}{FVIII(t)+IF_{VIII,50}} \right)$$

Where h(t) is the instantaneous risk of bleeding, $\lambda\cdot e^{\gamma\cdot\left( t-1 \right)}$ is the basal risk in absence of FVIII concentrations, FVIII(t) is the FVIII concentration at time t, IFVIII50 is the FVIII concentration at which the risk-to-bleed is halved.

The association between bleeding risk and FVIII concentration was assumed to be time invariant and identical independent of the FVIII product. Hence, differences in bleeding between BAY 81-8973 and rAHF-PFM were only due to differences in FVIII concentration time profiles. The proportions of different types of bleeds were taken from the existing literature. In the base case analysis, it was assumed that 38% of all spontaneous and traumatic bleeds occurred in the joints (5) and that 0.20% of all bleeds were major (i.e. life-threatening and required hospitalization (6). Patients in each bleeding per-year category accumulated joint bleeds over time, and based on the cumulative value, the Pettersson score was determined.

**Table S1. BAY 81-8973** **and rAHF-PFM PK parameters(7)**

| **Parameter** | **Units** | **Estimate** | **Description** |
| --- | --- | --- | --- |
| **CL_BAY_** | dL/h | 1.51 | Clearance of BAY 81-8973 |
| **V1_BAY;rAHF-PFM_** | dL | 23.6 | Central volume of distribution BAY 81-8973 and rAHF-PFM |
| **Q_BAY;fAHF-PFM_** | dL/h | 1.59 | Intercompartmental clearance of BAY 81-8973 and rAHF-PFM |
| **V2_BAY_** | dL | 5.35 | Peripheral volume of distribution of BAY 81-8973 |
| **IIV_Cl_** | % | 27.2 | Intraindividual variability in clearance |
| **IIV_V1_** | % | 8.0 | Intraindividual variability in central volume of distribution |
| **ΔCL_rAHF-PFM_** | % | 47.8 | Change in clearance for rAHF-PFM compared with BAY 81-8973 |
| **ΔV2_rAHF-PFM_** | % | 86.6 | Change in peripheral volume of distribution for rAHF-PFMcompared with BAY 81-8973 |

rAHF-PFM: antihemophilic factor (recombinant) plasma/albumin-free method

**Figure S1. Cost-effectiveness scatterplot**

1. Shah A, Solms A, Garmann D, Katterle Y, Avramova V, Simeonov S, et al. Improved Pharmacokinetics with BAY 81-8973 Versus Antihemophilic Factor (Recombinant) Plasma/Albumin-Free Method: A Randomized Pharmacokinetic Study in Patients with Severe Hemophilia A. Clinical pharmacokinetics. 2017;56(9):1045-55.

2. [Chinese guidelines on the treatment of hemophilia (version 2020)]. Zhonghua xue ye xue za zhi = Zhonghua xueyexue zazhi. 2020;41(4):265-71.

3. Abrantes J, Solms A, Garmann D, Nielsen E, Jonsson S, Karlsson M, editors. Integrated modelling of factor VIII activity kinetics, occurrence of bleeds and individual characteristics in haemophilia A patients using a full random effects modelling (FREM) approach. PAGE meeting; 2018; Montreux, Switzerland.

4. Saxena K, Lalezari S, Oldenburg J, Tseneklidou-Stoeter D, Beckmann H, Yoon M, et al. Efficacy and safety of BAY 81-8973, a full-length recombinant factor VIII: results from the LEOPOLD I trial. Haemophilia : the official journal of the World Federation of Hemophilia. 2016;22(5):706-12.

5. Tarantino MD, Collins PW, Hay CR, Shapiro AD, Gruppo RA, Berntorp E, et al. Clinical evaluation of an advanced category antihaemophilic factor prepared using a plasma/albumin-free method: pharmacokinetics, efficacy, and safety in previously treated patients with haemophilia A. Haemophilia : the official journal of the World Federation of Hemophilia. 2004;10(5):428-37.

6. Witmer C, Presley R, Kulkarni R, Soucie JM, Manno CS, Raffini L. Associations between intracranial haemorrhage and prescribed prophylaxis in a large cohort of haemophilia patients in the United States. British journal of haematology. 2011;152(2):211-

7. Shah A, Delesen H, Garger S, Lalezari S. Pharmacokinetic properties of BAY 81-8973, a full-length recombinant factor VIII. Haemophilia : the official journal of the World Federation of Hemophilia. 2015;21(6):766-71.
